# Multivariate meta-analysis: Potential and promise

**DOI:** 10.1002/sim.4172

**Published:** 2011-01-26

**Authors:** Dan Jackson, Richard Riley, Ian R White

**Affiliations:** a MRC Biostatistics UnitCambridge, U.K; b Department of Public Health, Epidemiology and Biostatistics, The Public Health Building, University of BirminghamBirmingham B15 2TT, U.K

**Keywords:** multivariate meta-analysis, random effects models, statistical software

## Abstract

The multivariate random effects model is a generalization of the standard univariate model. Multivariate meta-analysis is becoming more commonly used and the techniques and related computer software, although continually under development, are now in place. In order to raise awareness of the multivariate methods, and discuss their advantages and disadvantages, we organized a one day ‘Multivariate meta-analysis’ event at the Royal Statistical Society. In addition to disseminating the most recent developments, we also received an abundance of comments, concerns, insights, critiques and encouragement. This article provides a balanced account of the day's discourse. By giving others the opportunity to respond to our assessment, we hope to ensure that the various view points and opinions are aired before multivariate meta-analysis simply becomes another widely used *de facto* method without any proper consideration of it by the medical statistics community. We describe the areas of application that multivariate meta-analysis has found, the methods available, the difficulties typically encountered and the arguments for and against the multivariate methods, using four representative but contrasting examples. We conclude that the multivariate methods can be useful, and in particular can provide estimates with better statistical properties, but also that these benefits come at the price of making more assumptions which do not result in better inference in every case. Although there is evidence that multivariate meta-analysis has considerable potential, it must be even more carefully applied than its univariate counterpart in practice. Copyright © 2011 John Wiley & Sons, Ltd.

## 1. Introduction

Now that meta-analysis is well established in medical statistics, it is perhaps easy to forget that, until relatively recently, its use has been considered controversial by the medical community 1, 2. In particular, Eysenck's provocative article, published in the *British Medical Journal* in 1994 3, still makes interesting reading today, and some might argue that the difficulties he identified have yet to be satisfactorily resolved. Issues like the quality of studies, nonlinear associations, and the debate between fixed and random effects meta-analyses, which Eysenck alludes to by referring to ‘Adding apples and oranges’, have subsequently received a great deal of attention and are points that anyone contemplating performing a meta-analysis should consider carefully. The second problem that Eysenck describes is that ‘effects are often multivariate rather than univariate’ and he notes, in the context of an example involving passive smoking, that meta-analysis ‘attempts a univariate type of analysis of a clearly multivariate problem’. We agree that medical studies often examine multiple, and correlated, outcomes of interest to the meta-analyst. A simple example is overall and disease-free survival.

The general problem is therefore to make inferences about correlated study effects, where each study estimates one or more of them and ideally provides the corresponding within-study covariance matrix. Not all studies may provide estimates of all effects of interest, so it is vitally important to handle missing data in a suitable way. We will describe the precise form of the multivariate random effects model in Section 3, and methods for fitting it in Section 4, but until then it is essential that the reader keeps the general problem firmly in mind. For a detailed account of the univariate methods that are extended here, see Normand's tutorial 4.

The variation in the studies' effects is separated into two components by the random effects model. The within-study variation refers to the variation in the repeated sampling of the studies' results if they were replicated, and the between-study variation refers to any variation in the studies' true underlying effects. Hence, we have both within- and between-study correlations in the multivariate random effects model. Within-study correlation occurs because different effects are calculated using the same set of patients. For example, if the effects of interest relate to desirable outcomes such as overall and disease-free survival status, then they will almost necessarily be positively correlated.

The between-study correlation allows the true underlying outcome effects to be correlated and hence the studies' effects to be more or less correlated than we would expect from the within-study variation alone. An obvious situation where the between-study correlation is important is the meta-analysis of diagnostic test accuracy. Here, within studies, the sensitivities and specificities are assumed to be independent because they are calculated using data from different individuals. Despite this, a negative correlation between these quantities across studies is likely 5 because studies that adopt less stringent criterion for declaring a test positive invoke higher sensitivities and lower specificities.

We assume that a ‘two-stage’ approach to analysis is adopted. At the first stage, (typically standard) analyses of each trial are performed, and estimates of parameters of interest are obtained; for example, in a survival study, the estimated hazard ratios of overall and disease-free survival. The within-study covariance matrices are also obtained at this stage, containing the variance of each effect and their covariances. These estimates are then combined at the second phase. If the estimates are obtained from published papers, as is typically the case, then a two-stage approach is necessary but if individual patient data (IPD) are available a one-stage approach is possible and may be preferable. One-stage methods for IPD random effects meta-analyses have been suggested for continuous 6, binary 7, ordinal 8 and time-to-event data 9. When the within-study model is relatively computationally complex, as is the case in survival modelling for example, and the data set is large, one-stage meta-analysis methods become computationally unfeasible 10 and a two-stage approach becomes necessary.

Considerable progress has recently been made in the development of multivariate meta-analysis and a tutorial paper 11 on multivariate meta-analysis and meta-regression appeared in *Statistics in Medicine* less than a decade later than Eysenck's article. This tutorial mainly focussed on the bivariate case where the outcome pairs are arm-specific measures. Hence, conditional on the study-specific true underlying measures, all effects are assumed to be independent. Although this special case is useful in some settings, applications have been found where this assumption is clearly implausible. More recently, investigations have examined the effect of misspecifying the within-study correlations 12, 13. In order to perform multivariate meta-analyses more generally, purpose-built software has been written to fit the multivariate random effects model 14 so that this can now be used routinely in conjunction with a variety of estimation methods. Hence, the weaponry is now firmly in place: all that has to be decided now is if, when and how to wield it. Multivariate meta-analysis has an abundance of potential and promise over its univariate counterpart. In particular, it can describe the associations between the estimates of effect in order to help make predictions about the true effects of a new study and provide estimates with better statistical properties, due to the borrowing of strength that it enables.

In order to raise awareness of the recent methodological developments, and the applications that motivated them, the authors of this article organized a one day ‘Multivariate Meta-Analysis’ event on 26th January 2010 at the Royal Statistical Society (RSS). The authors initially presented the theory, and the applications followed. (Diagnostic tests: Roger Harbord, Theo Stijnen. Multiple parameter models: Stephen Kaptoge, Ben Armstrong and Antonio Gasparrini, Dan Jackson. Selective outcome reporting:Paula Williamson.) This meeting resulted in considerable enthusiasm and encouragement but concerns and issues were also raised and we felt it timely to provide a balanced account of the discourse of the meeting. Riley 13 notes that, with the exception of diagnostic test studies, ‘multivariate meta-analysis methods are rarely used by practitioners in systematic reviews’. Hence, if the concerns outweigh the benefits, it may not be too late to stifle multivariate meta-analysis in the way that Egger and Smith 2 suggested that some may think meta-analysis *per se* should have been as recently as 1997.

In this article we proceed as follows. In Section 2 we describe the areas of application that motivated multivariate methods. In Section 3 we discuss the multivariate random effects model and its assumptions. In Section 4 we describe the estimation methods that have been developed. In Section 5 we apply the methods to our example data sets and discuss the advantages and limitations of the multivariate methods in relation to these. In Section 6 we tackle perhaps the greatest practical difficulty: handling the (frequently unknown) within-study correlations. We conclude our article with a discussion, which is followed with invited commentaries from some of those present at the RSS meeting and others with an interest in meta-analysis.

## 2. Areas of application

The need for multivariate meta-analysis methodology has been driven by a variety of applications and in this section we describe some of these. In addition to the areas of medicine represented by our examples, applications have included education 15–18, dentistry 19, 20, marketing 21, surrogate outcomes 22, 23 and genetic epidemiology 24. The types of data that have been meta-analysed multivariately include survival 25, binary 5, ordinal 26, continuous 16 and longitudinal 27. We now describe four general areas where we regard multivariate meta-analysis to have been particularly successful.

### 2.1. Diagnostic test meta-analysis

Perhaps the most common medical application of multivariate meta-analysis is the bivariate meta-analysis of studies of diagnostic test accuracy 5, 28. Here, studies provide either the numbers of false and true, positive and negatives or estimates and standard errors of their sensitivity and specificity. As explained above, since these values are calculated from the true negative and true positive patients, respectively, the within-study correlations are zero. Diagnostic test studies are often small, and effects can be very large, so the use of binomial distributions for the within-study distributions is generally recommended 26, 29. This replaces the bivariate normal model within studies in equation (1) below with two independent intercept only logistic regressions. Purpose-built software 30, 31 is now in place to perform this kind of analysis.

This use of the bivariate random effects model for meta-analysis is perhaps especially appealing due to the pioneering work of Harbord *et al.* 32 who show that this is, under a wide range of circumstances, equivalent to the Hierarchical Summary Receiver Operating Characteristic (HSROC) model 33. Alternative models are also possible; a Poisson-correlated gamma frailty model 34, a trivariate model 35 and a Bayesian approach using Laplace approximations 36 have recently been developed and we expect further methodological development.

Our first example data set is a meta-analysis of prognostic test studies and is taken from Kertai *et al.* 37. The data structure is the same as a diagnostic test meta-analysis. The data are shown in Table I, where the true and false positives and negatives are from 7 studies of the sensitivity and specificity, which provide the two effects of interest, of exercise electrocardiography for predicting cardiac events in patients undergoing major vascular surgery.

**Table I:** Example 1: Estimates from 7 studies of sensitivity and specificity of measurement of exercise electrocardiography for predicting cardiac events in patients undergoing major vascular surgery

Study	True positives	False negatives	True negatives	False positives
1	8	1	79	32
2	1	0	10	6
3	2	1	78	24
4	1	0	32	41
5	3	4	44	9
6	2	0	44	2
7	2	0	93	48

### 2.2. Multiple effects in randomized controlled trials or observational studies

In any context where clinical trials or observational studies report more than a single outcome of interest, multivariate meta-analysis may be used. This presents an additional challenge not present in diagnostic testing because the within-study correlations must also be available. Despite this, multivariate meta-analysis has been successfully applied in this setting 38–40.

Our second and third examples are of this kind. A representative selection of the studies' results from the second example are shown in Table II and the full data set is available from the authors on request. This is similar to the example used by Riley 13 but here we include 73 observational studies that examine two effects, overall and disease-free survival. These studies assess the prognostic value of up to two factors, MYCN and Chromosome 1p, in patients with neuroblastoma. Patients either have ‘high’ or ‘low’ levels of MYCN and either Chromosome 1p presence or deletion. It is thought that patients with high levels of MYCN and Chromosome 1p deletion have worse prognosis. Studies provide up to four estimates of effect, each of which is an estimated unadjusted log hazard ratio of survival, either of the high relative to the low level group of MYCN, or Chromosome 1p deletion to its presence. Standard errors of all the various estimates are given but the within-study correlations are unknown to the authors. These tumour markers are thought to be highly correlated 41 and overall and disease-free survival are naturally likely to be highly positively correlated.

**Table II:** Example 2: Estimated unadjusted log hazard ratios from 73 studies

Study	*Y*_1_	*Y*_2_	*Y*_3_	*Y*_4_	*s*_1_	*s*_2_	*s*_3_	*s*_4_
1			1.31			0.82		
2			3.33			0.71		
3			2.37			0.72		
4	1.64		1.54		0.51		0.52	
5			2.07			0.69		
6	-0.11		-0.14		0.67		0.81	
7	1.46	0.80	1.51	0.95	0.41	0.44	0.48	0.52
·	·	·	·	·	·	·	·	·
·	·	·	·	·	·	·	·	·
73			0.91			0.66		

The variables *Y*_1_ and *Y*_2_ denote the log hazard ratio for disease-free survival for high to low MYCN, and the deletion to the presence of Chromosome 1p markers, respectively. *Y*_3_ and *Y*_4_ denote the corresponding overall survival log hazard ratios. *s*_1_ to *s*_4_ denote these variables' within-study standard errors. 34, 8, 50 and 10 studies report *Y*_1_ to *Y*_4_, respectively. The within-study correlations are unknown.

Our third example 42 is a meta-analysis that summarizes the existing evidence about whether the presence of mutant p53 tumour suppressor gene is a prognostic factor for patients presenting with squamous cell carcinoma arising from the oropharynx cavity. Unadjusted estimates of log hazard ratios of mutant p53 to normal p53, and their standard errors from 6 observational studies are shown in. Here, *Y*_1_ denotes the log hazard ratio for disease-free survival and *Y*_2_ denotes the log hazard ratio for overall survival. Only 3 studies provide estimates for disease-free survival. The within-study correlations are again unknown to the authors but are expected to be highly positively correlated.

### 2.3. Multiple parameter models for exposure in observational studies

The multivariate methods lend themselves to the meta-analysis of observational IPD. Here, we wish to pool information across studies for exposure parameters that represent effects of particular interest. Typically, we include more covariates in our within-study models that we wish to adjust inferences for.

Our fourth example is of this kind 43. The aim was to describe the association between fasting glucose level and cardiovascular disease and seven groups were formed for this purpose. Upon removing studies with fewer than 11 coronary vascular disease events, we have six estimated log hazard ratios from each of 39 studies. Each of these hazard ratios is for groups of participants relative to the ‘baseline group’, i.e. those with no known diabetes at baseline, and a fasting glucose of 3.9–5.6 mmol/L (). The IPD was used to fit the proportional hazards model to each study separately which was stratified, where appropriate, by sex and study group, and adjusted for age, smoking status, BMI and systolic blood pressure. Covariance matrices for the estimates from every study are available, as obtained from the observed information matrix when fitting the proportional hazards model to each study, and hence the within-study correlations are known for this example.

### 2.4. ‘Network’ meta-analysis

The multivariate methods also lend themselves to ‘network meta-analysis’ 44–46, indeed it is hard to imagine such analyses in anything other than a multivariate setting. Here, studies simultaneously compare multiple treatments and so provide results for multiple treatment groups. Just as in the meta-analysis of diagnostic test accuracy studies, this scenario presents something of a special case and therefore its own issues and difficulties. We will not discuss this particular application further in this article but we suspect that this type of analysis will continue to motivate the development of multivariate methods.

## 3. Model and assumptions

### 3.1. The within-study model

We denote the vector of effects (or estimates) for the *i*th study as **Y**_*i*_. The entries of **Y**_*i*_ may be correlated and it is assumed that within each study


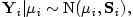
(1)

where N denotes a *multivariate* normal distribution, µ_*i*_ is the true underlying effect for the *i*th study and **S**_*i*_ is the covariance matrix of **Y**_*i*_. The matrices **S**_*i*_ are referred to as the within-study covariance matrices; their entries are estimated in practice using the IPD for each study separately but regarded fixed and known when pooling the results to make inferences. The within-study variances (the diagonal entries of **S**_*i*_, whose square roots give the within-study standard errors are shown in Tables II and III) are typically obtained in the same manner as in the univariate case. We return to the issue of estimating the within-study correlations, from which the off-diagonal entries of **S**_*i*_ can be obtained, in Section 6. Model (1) is simply the usual multivariate normal approximation to the studies' estimated effects and hence is relatively uncontroversial provided the study sizes are large enough. Other within-study models, such as logistic regressions for binary data, may also be used. For example, Chu *et al.* 35 model all the three outcomes in this manner but non-normal within-study distributions add to the computational demands.

**Table III:** Example 3: Estimated log hazard ratios from 6 studies

Study	*Y*_1_	*Y*_2_	*s*_1_	*s*_2_
1	−0.58	−0.18	0.56	0.56
2		0.79		0.24
3		0.21		0.66
4	−1.02	−0.63	0.39	0.29
5		1.01		0.48
6	−0.69	−0.64	0.40	0.40

The variable *Y*_1_ denotes the log hazard ratio for disease-free survival for a mutant to normal p53 gene. *Y*_2_ denotes this quantity for overall survival. *s*_1_ and *s*_2_ denote these variables' within-study standard errors. The within-study correlations are unknown.

### 3.2. The between-study model

The multivariate random effects model allows the µ_*i*_ to vary from one study to the next and further assumes that


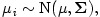
(2)

where µ is the (overall) treatment effect vector and Σ is the between-study covariance matrix. We interpret µ as the average effect from a normal distribution of study effects. We regard Σ as being unstructured but simplifications are possible: for example, all between-study correlations, or all between-study variances, could be assumed to be the same.

In the presence of missing study effects, there must be enough estimated effects, and combinations of these within studies, to make Σ identifiable. We assume throughout that this is so. If all entries of Σ are determined to be zero, then the model reduces to a fixed effects (common mean for effects across all studies) model. For those who prefer to fit fixed effects models, or reduced random effects models with fewer heterogeneity parameters (for example, a random effects model where all univariate between-study variances are identical), when these appear to describe the data reasonably well, Ritz *et al.* 47 provide hypothesis tests. We find the assumption that there is no between-study variation in any of the effects of interest particularly implausible in the multivariate setting and adopt a random effects approach here.

The model for the random effects (2) is harder to justify than (1). With small numbers of studies the normality assumption is difficult to check empirically and we can only tacitly invoke the Central Limit Theorem by assuming that the random effects are the sum of several factors. Rather than being entirely innocent, (2) makes some important assumptions by assuming between-study normality. These include:

1. A multivariate linear between-studies regression.
2. A further consequence of the assumed normality is a constant between-studies covariance matrix, where the conditional variances of all components of the random effect are also constant.
3. A normal distribution for the random effect implies that this is symmetrical and does not allow for a distribution with heavy or light tails.

When all, or the majority of, studies provide all effects then these assumptions are not so worrying. When a relatively large number of studies do not provide all effects, assumptions 1–3 become more of a concern, as the borrowing of strength discussed below depends on the distributional relationships between the observed and unobserved effects. For example, an outlying trial result on one outcome could be very influential for the inferences for both this and other effects, because of the assumed distributional form of the random effect and the linear regressions. If separate univariate meta-analyses are conducted such an outlying result could still be influential 48, but only for the inferences relating to the particular outcome the outlier relates to. The extent to which model (2) drives inferences, and how this depends on the nature of the data and the dimensionality of the meta-analysis, is currently poorly understood. Alternative random effects distributions have been considered in the univariate case 48, 49.

### 3.3. The marginal model

Marginally, this provides the conventional multivariate random effects meta-analysis model


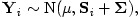
(3)

where the **Y**_*i*_ are further assumed to be independent because they come from separate studies. For any studies that provide only some effects, the model for the studies' results are taken as their submodel from (3). The conventional univariate random effects model is simply the normal distribution of one study effect. Furthermore, the collection of univariate models for each outcome is model (3) with all off-diagonal entries of all covariance matrices set to zero. Our aim is to estimate µ and Σ. Once 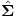 has been calculated, the estimated between-study correlations can be obtained directly as the appropriate entry of 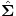 divided by the corresponding between-study standard deviations, which are obtained as the square roots of the diagonal entries. Estimated between-study standard deviations are given for some of the examples that follow because they provide additional insight into the model fit.

## 4. Estimation

A variety of approaches for fitting the random effects model for meta-analysis have been developed, and these can be divided into two categories: those that effectively use the estimated between-study covariance matrix as if it were the true value when making inferences about the treatment effect, which we regard as the standard procedure because it is simpler to apply, and those that do not 47. We describe a variety of methods of estimating the between-study covariance matrix below. Assuming all studies provide all effects, the pooled estimates 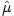 are given in terms of 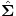 by


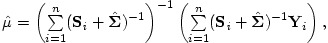
(4)

where *n* is the number of studies.

### 4.1. The standard procedure for making inferences about the effects

As noted above, the standard procedure involves approximating the true between-study variance with 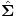 when making inferences about the treatment effect. The approximation that underlies these methods is justifiable provided that the number of studies is sufficiently large. Jackson 50 provides guidelines concerning how many studies are required in the univariate setting but it remains an open question as to how many studies are needed as the dimension of the meta-analysis increases. Concerns have also been raised about approximating the within-study variances in the conventional way in the univariate setting. This is not usually to directly question the validity of the conventional approximation (1) but rather to emphasize that the weights allocated to studies are functions of these variances and any uncertainty in the variance structure transfers to the weights and hence to the statistical properties of the estimates. Whether this is more, or less, of a concern in the multivariate setting is also currently poorly understood.

This standard approach is attractive because, assuming all studies provide all effects, estimates are approximately normally distributed with covariance matrix


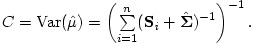
(5)

Hence, univariate and joint confidence regions can be obtained. For example, an approximate (1−α) per cent confidence interval can be obtained for µ_1_ as 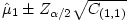, where *Z*_α/2_ denotes the α/2 percentile of a normal distribution and *C*_(*i, j*)_ denotes the entry in the *i*th row and *j*th column of *C*. The use of quantiles from the *t* distribution for making inferences, rather than the standard normal, has been suggested 51. Alternatively, if likelihood-based methods have been used, standard errors of estimates can be obtained from either the observed or expected Fisher information matrix. Between-study variance estimates frequently lie at the edge of their parameter space which presents difficulties when obtaining standard errors for all parameters in this way in practice 10 and this can also result in other statistical issues 51.

If some studies have missing effects then, assuming that these are missing at random and for computational convenience, such studies can be incorporated into the matrix solutions (4) and (5) by allocating very large within-study variances to these missing observations and within-study correlations of 0. This replaces missing effects with estimates with negligible weight and information. Alternatively, upon taking Σ as fixed, the log-likelihood is perfectly quadratic and any variation of the usual asymptotic maximum likelihood procedures can be used to provide inference for the treatment effect.

The main statistical difficulty lies in estimating the between-study covariance matrix Σ. As the estimation of this is typically fairly imprecise for examples with small numbers of studies, the resulting statistical procedures may not perform well as can be seen in some simulation studies of Jackson *et al.* 52. A variety of estimation methods have been proposed.

#### 4.1.1. Maximum likelihood estimation

As the likelihood is the product of normal densities it can be maximized numerically to simultaneously give estimates of the entries of Σ, subject to the constraint that this matrix is positive semi-definite, and 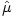. In high dimensions, probably the easiest way to ensure that 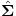 is positive semi-definite is to perform the maximization in terms of its Cholesky decomposition, Σ = **LL**^T^, and back transform to obtain 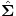. The multivariate random effects model is invariant to linear transformations of the data and hence so are likelihood-based inferences.

#### 4.1.2. Restricted maximum likelihood (REML)

It is more usual to estimate the entries of the between-study covariance matrix using REML. The restricted likelihood is a function of the variance components only (i.e. not µ) and REML helps to correct for the downward bias of maximum likelihood estimates of variance components. Estimation is performed by maximizing a special case of the expression λ_REML_ given by Jennrich and Schluchter 53, p. 812 (again subject to the constraints that the between-study covariance matrix is positive semi-definite):


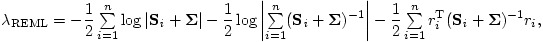


where the *r*_*i*_ denote the residuals and 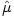 is obtained from (4).

The main difficulty presented by these likelihood-based methods for estimating the between-study covariance matrix is their computational intensity as the dimension of the meta-analyses increases.

#### 4.1.3. The method of moments

The univariate method of DerSimonian and Laird 54 has recently been extended to the multivariate scenario 52. An easily computed matrix generalization of Cochran's heterogeneity statistic is defined, whose expected entries are each linear functions of just one of the entries of Σ. Moment estimates of each entry are obtained by solving linear equations and 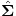 can be ‘truncated’, using standard matrix operations, so that it is positive semi-definite, as explained by Jackson *et al.* 52. This is easily the least computationally intensive method for multivariate meta-analysis and, since the procedure for estimating 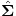 relies solely on moments arguments, an estimate of the between-study variance can be obtained without the assumption of between-study normality. Hence, a valid, but not optimal, meta-analysis can be performed without assumption (2). However, the nature of the pooling in (4) is still equivalent to a multivariate linear regression and the proposed ‘Cochran's heterogeneity matrix’ is not invariant to linear transformations of the data.

### 4.2. Alternative procedures that allow for the uncertainty in the between-study covariance matrix

In addition to these procedures, more computationally intensive alternatives are possible that allow for the uncertainty in the between-study covariance matrix and hence may perform better. For example, Kenward and Roger 55 give small sample approximations for REML whose use in the context of meta-analysis awaits investigation.

#### 4.2.1. Profile likelihood

The use of profile likelihood in meta-analysis was established by Hardy and Thompson 56 and used by the Fibrinogen Studies Collaboration 10 in the bivariate setting. In the univariate scenario it outperforms the standard procedures when the sample size is small, in terms of the actual coverage of nominal 95 per cent confidence intervals 57. A difficulty for the routine use of the profile likelihood is that very large numbers of numerical maximizations are needed which becomes prohibitive as the dimension of the meta-analysis increases.

#### 4.2.2. Bayesian analyses

Complex Bayesian analyses are now computationally feasible due to the advent of MCMC methods which WinBUGS 58 in particular has popularized. By placing ‘vague’ priors on all parameters, analyses that approximate likelihood-based inferences can, in principle, be obtained. For example, Nam *et al.* 59 use WinBUGS to perform Bayesian multivariate meta-analyses. A recent investigation shows that alternative and apparently vague priors can produce markedly different results in the univariate setting 60 and this situation is likely to worsen as the dimension of the analysis, and hence the number of parameters, increases 61. Hence, we advocate caution when using this type of approach and sensitivity analysis to the choice of prior distributions is highly recommended.

The multivariate setting provides the additional challenge of placing vague priors that ensure that the between-study correlation matrix is positive semi-definite; at our RSS meeting it was suggested that a uniform prior be placed on log(ρ/(1−ρ)) in the bivariate case, rather than on the correlation ρ directly. In high dimensions perhaps the easiest way to attempt to use a vague prior for the between-study covariance matrix is to use a Wishart prior 62. Because of the flexibility of modelling, MCMC has become the *de facto* method for the analysis of network meta-analyses where ‘incoherence’ or ‘inconsistency’ is modelled. A Bayesian approach also facilitates incorporating external evidence via informative priors if desired, and may be particularly useful to help estimate the between-study correlation.

### 4.3. Software

The Stata program *mvmeta1* is used throughout the following section to obtain the results for our example data sets. *mvmeta1* is an updated version of *mvmeta* 14 which can perform multivariate meta-regression and is available from the third author's website. It is hoped that this program will shortly be published as *mvmeta* version 2. All the three estimation methods described in Section 4.1 have been implemented in both *mvmeta* and *mvmeta1*. The Stata program *metandi* 30 was used to analyse our first example bivariately using maximum likelihood. SAS' *PROC NLMIXED* and *METADAS* are however fully viable alternatives for performing multivariate analyses.

## 5. Illustrated examples of the advantages and limitations of multivariate meta-analysis

In this section we describe the advantages and potential limitations of multivariate meta-analysis, and use the four examples introduced in Section 2 to illustrate the key concepts. For each of example data sets 2–4 (Tables II and III), we used the conventional multivariate random effects model described in Section 3. Estimation was performed using REML, and the method of moments for comparison. For example 1, we again fitted model (2) between-studies (assuming bivariate normality between studies for logit-sensitivity and logit-specificity), but within studies we modelled the binomial nature of the data directly, as described elsewhere 35. This was undertaken using maximum likelihood.

### 5.1. Summary of results

The results of the meta-analyses of examples 1–4 are shown in Tables V–VIII. These tables are set out differently because each example has different dimensions and presents its own issues and difficulties. Table V shows that univariate and bivariate analyses of our prognostic test studies example are in reasonable agreement and suggest that this test has only moderate value. Tables VI and VII show the results using REML and the method of moments, assuming various values for the unknown within-study correlations, for our examples involving multiple effects as described in Section 2.2. Table VI shows that the average log hazard ratio estimates are significantly greater than zero, and hence Chromosome 1p and MYCN have prognostic value for both disease-free and overall survival. In Table VII, p53 is not prognostic for overall survival (average log hazard ratio is not significantly different from zero) but the prognostic value for disease-free survival is debatable, as the significance of the average log hazard ratio clearly depends on whether univariate or bivariate meta-analysis is used, and also the procedure used when estimating the bivariate model (this issue is discussed further below). Finally, Table VIII shows the results for our final example introduced in Section 2.3. There is strong evidence that groups B to F (as defined in Table IV) are all at a higher risk of a cardiovascular event compared with the baseline group, as the average log hazard ratio parameter estimates shown are significantly greater than zero.

**Table IV:** Example 4: The seven exposure groupings used

Group	Description
Baseline	No known history of diabetes. Fasting glucose 3.9–5.6 mmol/L
A	No known history of diabetes. Fasting glucose less than 3.9 mmol/L
B	No known history of diabetes. Fasting glucose 5.6–6.1 mmol/L
C	No known history of diabetes. Fasting glucose 6.1–7 mmol/L
D	No known history of diabetes. Fasting glucose greater than 7 mmol/L
E	Known history of diabetes. Fasting glucose less than 7 mmol/L
F	Known history of diabetes. Fasting glucose more than 7 mmol/L

Thirty-nine studies, with 11 or more cardiovascular disease events, provide all the six estimates of the log hazard ratio of groups A–F, relative to the baseline group, and all corresponding within-study variances and correlations.

**Table V:** Results for example 1 using maximum likelihood

	Univariate	Bivariate
Logit-sensitivity	1.41 (0.76) [0.79]	1.49 (0.78) [0.90]
Logit-specificity	1.03 (0.33) [0.79]	1.02 (0.31) [0.76]

Standard errors of estimates are in parentheses and the estimated between-study standard deviations are shown in square brackets.

**Table VI:** Results for example 2

	Univariate	ρ = 0	ρ = 0.3	ρ = 0.7	ρ = 0.95
*REML*					
µ_1_	1.58 (0.14) [0.57]	1.58 (0.13) [0.59]	1.58 (0.12) [0.57]	1.59 (0.11) [0.56]	1.57 (0.10) [0.56]
µ_2_	1.33 (0.29) [0.67]	1.29 (0.26) [0.82]	1.25 (0.26) [0.75]	1.18 (0.28) [0.75]	1.01 (0.29) [0.92]
µ_3_	1.69 (0.13) [0.61]	1.73 (0.13) [0.70]	1.72 (0.13) [0.68]	1.71 (0.12) [0.67]	1.70 (0.11) [0.65]
µ_4_	1.26 (0.23) [0.47]	1.17 (0.22) [0.64]	1.15 (0.22) [0.64]	1.15 (0.20) [0.62]	1.13 (0.16) [0.70]
*MM*					
µ_1_	1.58 (0.14) [0.60]	1.60 (0.14) [0.70]	1.59 (0.13) [0.66]	1.58 (0.12) [0.61]	1.58 (0.12) [0.60]
µ_2_	1.33 (0.28) [0.64]	1.28 (0.29) [0.78]	1.27 (0.27) [0.74]	1.27 (0.25) [0.70]	1.30 (0.22) [0.67]
µ_3_	1.69 (0.13) [0.65]	1.72 (0.13) [0.72]	1.71 (0.13) [0.69]	1.71 (0.12) [0.66]	1.71 (0.12) [0.65]
µ_4_	1.26 (0.24) [0.49]	1.25 (0.27) [0.72]	1.24 (0.25) [0.68]	1.22 (0.22) [0.61]	1.20 (0.19) [0.57]
Max LL		−299.17	−296.03	−290.71	−285.72

‘REML’ indicates that REML has been used (top half of the table) and ‘MM’ indicates that the method of moments has been used. The parameters µ_*i*_ are the log hazard ratios corresponding to the effects shown in Table II and ρ denotes the common assumed within-study correlation. Standard errors of estimates are in parentheses and the estimated between-study standard deviations are shown in square brackets. Max LL denotes the maximum log-likelihood obtained using the within-study correlations shown in a multivariate meta-analysis.

**Table VII:** Results for example 3

	Univariate (REML)	ρ = 0.7 (REML)	ρ = 0.95 (REML)	Univariate (MM)	ρ = 0.7 (MM)	ρ = 0.95 (MM)
µ_1_	−0.80(0.25)	−0.32 (0.42)	−0.28 (0.31)	−0.80(0.25)	−0.77(0.26)	−0.76 (0.26)
	[0]	[0.46]	[0.41]	[0]	[0.10]	[0.15]
µ_2_	0.09(0.31)	0.09(0.31)	0.10 (0.31)	0.09 (0.34)	0.07 (0.34)	0.06(0.34)
	[0.64]	[0.63]	[0.62]	[0.70]	[0.71]	[0.71]
κ		1	1		−1	−1
Max LL		−8.59	−7.51		−8.59	−7.51

The parameters µ_*i*_ are the log hazard ratios corresponding to the effects shown in Table III and ρ denotes the common assumed within-study correlation. ‘REML’ indicates that REML has been used and ‘MM’ indicates that the method of moments has been used. Standard errors are in parentheses and the estimated between-study standard deviations which correspond to the parameter in question are shown in square brackets. Max LL denotes the maximum log-likelihood obtained using the within-study correlations shown in a multivariate meta-analysis and κ denotes the estimated between-study correlation.

**Table VIII:** Results for example 4

	Univariate	REML	MM
A	0.09 (0.04) [0]	0.06 (0.07) [0.07]	0.05 (0.05) [0.09]
B	0.08 (0.03) [0]	0.09 (0.03) [0.04]	0.10 (0.03) [0.07]
C	0.11 (0.04) [0]	0.14 (0.05) [0.10]	0.14 (0.05) [0.12]
D	0.56 (0.07) [0.16]	0.58 (0.10) [0.23]	0.56 (0.07) [0.20]
E	0.46 (0.08) [0.18]	0.46 (0.10) [0.33]	0.43 (0.08) [0.27]
F	0.86 (0.10) [0.39]	0.87 (0.10) [0.41]	0.87 (0.09) [0.41]

Estimates are log hazard ratios for each group in Table IV relative to the baseline group. Standard errors are in parentheses and estimated between-study standard deviations which correspond to the parameter in question are shown in square brackets. ‘REML’ denotes restricted maximum likelihood estimation and ‘MM’ denotes that the multivariate method of moments has been used.

### 5.2. Advantages of multivariate meta-analysis

#### 5.2.1. We obtain estimates for all effects within a single modelling framework

It is more elegant to perform a single multivariate meta-analysis than many univariate ones. This advantage is demonstrated well by our fourth example (Table VIII). The multivariate meta-analysis results, using either REML or moments, give reasonably similar parameter estimates to the separate univariate analyses, but these are simultaneously provided in a single analysis.

#### 5.2.2. We can describe and utilize the relationship between the multiple effects

**Figure 1:**
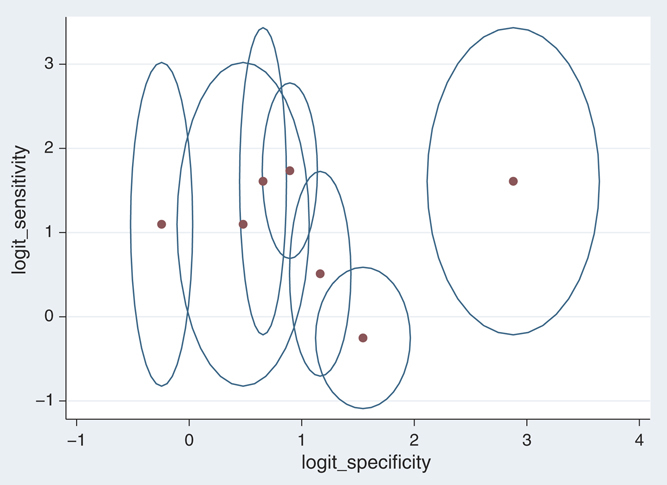
Bubbleplot of the 7 studies that comprise example 1. The bubbles show 50 per cent study-specific confidence regions based on normal within-study approximations.

This advantage is nicely demonstrated by our first example (Tables I and V). Figure 1 suggests that there may be a negative relationship between sensitivity and specificity across studies but this is hard to assess from a visual inspection of the data. This is reflected by the large and negative estimate of −0.95 for the between-study correlation. In comparison, a univariate analysis naively assumes the correlation is zero, which leads to slightly different parameter estimates (Table V).

**Figure 2:**
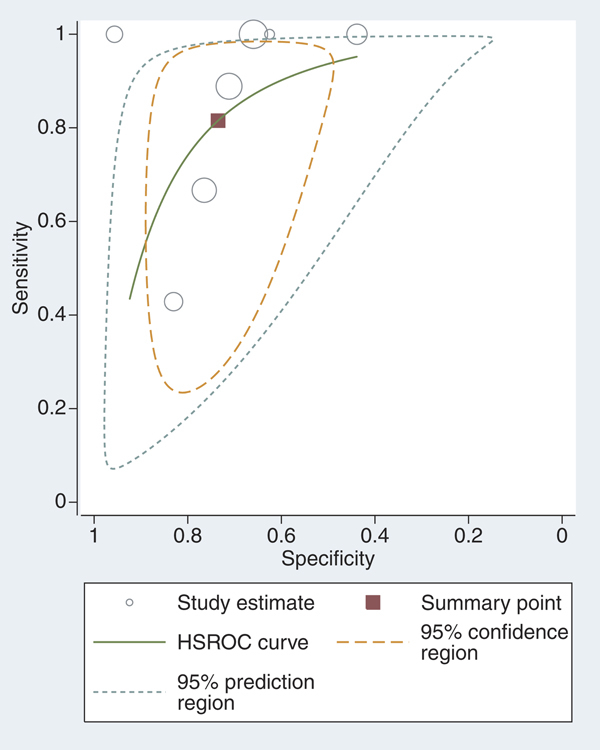
Plot of fitted model to example 1 from *metandi*.

The utilization of between-study correlation here allows the appropriate calculation of a joint confidence region around the pooled sensitivity and specificity pair, as shown in Figure 2. Similarly, it allows a joint prediction region 63 for the true sensitivity and specificity in an individual study setting. For a detailed explanation of how this region is obtained, see Harbord and Whiting 30. Furthermore, as Hand 64 points out, weighted sums of the estimated sensitivity and specificity are typically used to assess the value of a test, but the properties of the resulting statistic depends crucially on the association between the estimates, and it is precisely this association that is ignored in separate univariate analyses.

In some situations one may wish to use multivariate meta-analysis to fit a line through the multiple effects of interest. For example, in our first example the summary ROC curve is derived by assuming a linear trend between logit-sensitivity and logit-specificity between studies (Figure 2), an idea recently extended when multiple thresholds are available for each study 26. Similarly, in meta-analysis of longitudinal data, where there is an effect of interest at each of a series of time-points, one may wish to model the trend in effect across the time-points. It is clearly important to account for the correlation between effects when modelling such a trend. For example, Jones *et al.* 27 show that univariate meta-analysis of longitudinal data, which ignores the correlation between time-points when fitting a line between them, leads to underestimated standard errors and overestimated treatment effects. One may also wish to estimate a function of the pooled effects. For instance, in example 2 we may want to subsequently estimate the difference in the overall survival hazard ratio between MYCN and Chromosome 1p, to assess which has more prognostic value. As estimates come from the same studies and are hence correlated, appropriate statistical procedures require a multivariate approach. For example, 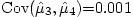 for the method of moments estimates and ρ = 0.95. We evaluate the standard error of 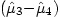 as 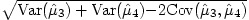 and obtain a statistically significant difference in the prognostic values. If the two corresponding univariate analyses are treated as if independent however then this significance is lost. For similar reasons, van Houwelingen *et al.* 11 use a bivariate meta-analysis to investigate the relationship between baseline risk and treatment effect.

#### 5.2.3. We obtain parameter estimates with better statistical properties

Parameter estimation is often superior in a multivariate meta-analysis than in a univariate meta-analysis, again as it utilizes the correlation between the endpoints of interest and thus each endpoint ‘borrows strength’ from the other related endpoints. Assuming that the between-study variance estimates are the same in multivariate and univariate analyses, Riley *et al.* 25 show analytically that the multivariate meta-analysis model of Equations (1) and (2) produces pooled estimates that have smaller standard errors than those from separate univariate models. The only situations where gains in precision cannot occur are when the within-study and between-study correlations are all zero, or when all studies provide all endpoints and the within-study variances of the same endpoint are identical 25.

The gain in efficiency of parameter estimates is most clearly demonstrated by our second example (Tables II and VI). There are many studies that only provide results for MYCN and not Chromosome 1p, so there is considerable opportunity for inferences concerning the effect of Chromosome 1p to borrow strength from MYCN. In this example, the within-study correlations are unknown but the various effects are either thought or known to be positively correlated as discussed in Section 2. Here, we have no IPD and in a sensitivity analysis we assume in turn that all within-study correlations are 0, 0.3, 0.7 and 0.95. The multivariate meta-analysis results (Table VI) show that as we increase the within-study correlation, we generally obtain more precise estimates. For example, assuming a within-study correlation of 0.95, the multivariate analysis using moments reduces the standard error by around 20 per cent for µ_2_ and µ_4_ compared with the univariate analyses.

The multivariate approach also improves estimation of the between-study variances, and thus borrowing of strength occurs for both the pooled estimates and the between-study variance estimates. For example, it has been shown that the multivariate model of Equations (1) and (2) gives a smaller mean-square error of the between-study variances than the univariate method 51. Similarly, in a multivariate analysis that models binomial data directly within studies followed by Equation (2), it has been shown that the mean-square error and also the downward bias of between-study variance estimates is reduced compared with the univariate approach^51^. Researchers can expect multivariate meta-analysis to produce, on average, pooled estimates with smaller standard errors and also mean-square errors. However, in an individual example the gain in precision also depends on the change in between-study variance estimates. For example, for group C in example 4, the standard error of the pooled log hazard ratio is actually greater in the multivariate meta-analyses. This is largely due to the positive estimated corresponding between-study variances from the multivariate analyses. The univariate analysis for group C estimates zero between-study variance (Table VIII) which results in a smaller standard error.

#### 5.2.4. We can obtain potentially different clinical conclusions compared with univariate meta-analysis

Conclusions from a multivariate meta-analysis may sometimes differ from those from univariate meta-analysis. For example, consider the comparison of group A to the baseline group in example 4 (Tables IV and VIII). The univariate meta-analysis gives a significant log hazard ratio of 0.09 (pooled hazard ratio = 1.09, 95 per cent confidence interval: 1.01–1.19); however, the multivariate meta-analysis gives a smaller non-significant log hazard ratio of 0.06 (using REML: pooled hazard ratio = 1.06, 95 per cent confidence interval: 0.92 to 1.22), now indicating no statistical evidence of a difference in cardiovascular event risk between group A and the baseline group. If alternative assumptions lead to markedly different conclusions then this is of interest and should be reported. This advantage is perhaps related to the advantage described in Section 5.2.; by providing all results in a single multivariate meta-analysis it is easier to compare the results from different analyses that make alternative assumptions.

#### 5.2.5. The multivariate methods have the potential to reduce bias due to partial reporting

In our third example (Tables III and VII), which relates to the prognostic ability of marker p53, overall survival results are available in all the 6 studies, but disease-free survival results are only available in the 3 studies. In univariate meta-analysis, one must assume for disease-free survival that the 3 available estimates reflect the evidence-base despite the missing data. This assumption is highly questionable because the log hazard ratio estimates are all negative in the three studies reporting both outcomes, but are all positive in those studies reporting only overall survival. Thus, due to the expected large correlation between overall and disease-free survival, there is a strong concern that the 3 missing disease-free survival estimates are also likely to be positive. A univariate meta-analysis is in danger of producing results biased in favour of negative log hazard ratios for disease-free survival.

A multivariate meta-analysis can utilize the correlation between overall survival and disease-free survival to borrow strength and reduce this problem. The univariate approach gives a pooled log hazard ratio for disease-free survival of −0.80 (hazard ratio = 0.45; 95 per cent confidence interval = 0.27 to 0.74), indicating there is large statistically significant evidence that patients with mutant p53 have a decreased event risk (Table VII). However, using REML and imputing within-study correlations (as they were not known) of either 0.7 or 0.95, in order to reflect the inevitable positive correlation between the estimates, the multivariate approach estimates a large between-study correlation of 1 and the inferences for disease-free survival borrow strength from the overall survival results, leading to a larger between-study variance estimate and a pooled log hazard ratio that is not statistically significant and much closer to the null (Table VII).

### 5.3. Potential limitations of multivariate meta-analysis

#### 5.3.1. Univariate meta-analysis is simpler and easier to understand

Separate univariate meta-analyses are more transparent and easier to understand than a multivariate method. A related argument is that we do not usually model effects from individual trials multivariately, so why should we attempt this in the context of meta-analysis?

#### 5.3.2. Multivariate meta-analysis can cause estimation difficulties

Sophisticated modelling is extremely difficult in meta-analysis without IPD. All we usually have are a handful of estimates and, if we are lucky, their standard errors. Multivariate meta-analysis often also requires within-study correlation estimates, but these are rarely available as in examples 2 and 3. In such situations sensitivity analyses, or some other approaches (see Section 6), are needed to limit this problem, which is not ideal.

Even when the within-study correlations are available, it is often difficult to estimate the between-study correlation and it is often estimated as 1 or −1, at the boundary of its parameter space, causing a slight upward bias in the between-study variance estimates 51. This estimation problem is evident in example 3 (Tables III and VII). There are just 3 studies that provide both outcomes, and the between-study correlation is imprecisely estimated as 1 using REML. Even more concerning, the method of moments estimation disagrees considerably and estimates it as −1. This causes large discrepancy between the method of moments and REML parameter estimates and the amount of borrowing of strength (Table VII), especially for disease-free survival which has a statistically significant pooled hazard ratio for moments but not for REML. Although a positive correlation between overall and disease-free survival makes more sense here, it is not clear which estimation method is more correct, if either, and additional data are required.

Some discrepancies between the method of moments and REML are also evident in the parameter estimates for Chromosome 1p in example 2, where REML provides smaller pooled estimates. The method of moments is a semi-parametric method for estimating the random effects, and it seems the stronger multivariate normality assumption of REML when estimating the between-study covariance matrix is leading to different conclusions and perhaps additional borrowing of strength.

#### 5.3.3. Additional assumptions are required by the multivariate methods

In a univariate meta-analysis the assumption that the random effects are normally distributed is hard to verify. In a multivariate meta-analysis, the multivariate normality assumption is even stronger and harder to verify. Furthermore, in the multivariate case, an implicit assumption is that the effects have a linear relationship between studies. It is hard to estimate nonlinear relationships with the few studies meta-analysis usually has available, but clearly the borrowing of strength will be influenced by this assumption. This may be particularly crucial when borrowing strength beyond the range of data for which an effect is available. For example, in example 3 the relationship between overall and disease-free survival p53 hazard ratio estimates is observable across those 3 studies that report both outcomes (Table III); however, in the other 3 studies for which only overall survival is available, the relationship between overall and disease-free survival hazard ratios is not observable and might differ. This is especially important for the p53 data, as the 3 studies providing both outcomes seem to disagree considerably with the other 3 studies. Further research on this issue is needed.

#### 5.3.4. Statistical properties of the individual parameter estimates are often only marginally improved

We were expecting our audience at our RSS meeting to be more impressed with the borrowing of strength within multivariate meta-analysis, and the more precise estimates it often brings. However, the enthusiasm level was rather underwhelming, even when the standard errors of pooled estimates dropped by around 10 to 30 per cent, as they do for many of the estimates in example 2 (Table VI). The view was that, in terms of the individual parameter estimates themselves, unless there is a large amount of missing data as in examples 2 and 3, the borrowing of strength may only be small and there may be little or no gain in precision for the pooled estimates. This was the conclusion of Sohn *et al.* 21 and also Simel and Bossuyt 65 after complete data comparison of univariate and multivariate meta-analysis results. Their conclusion is exemplified by the hazard ratio estimates and their precision for groups B to F in Table VIII.

While we agree gains in statistical properties are often only small, particularly for complete data, it is important to note here that even small changes in estimates and their precision can change statistical significance and clinical conclusions, as discussed earlier for group A in example 4 (Table VIII).

#### 5.3.5. Publication biases might be exacerbated

A further potential difficulty is publication and related biases 66 and the arguments in Section 5.2.5 implicitly assume data are missing at random. A natural concern is that the routine use of multivariate meta-analysis will encourage the joint analysis of both primary and secondary effects. If the secondary effects are prone to publication bias, where data are missing not at random, then the inferences for the both primary and secondary effects will be biased. Addressing the possibility of publication bias is perhaps especially important and difficult in the multivariate setting because the multivariate structure of the data has the potential to allow biases to manifest themselves in less direct and more subtle ways. See Jackson *et al.* 67 for the full analysis of a case study which exemplifies this issue.

## 6. Handling unknown within-study correlations

Perhaps the greatest difficulty applying the multivariate meta-analysis model in practice is that the within-study correlations are required by the model and are typically unknown as in our second and third examples.

Recall from Section 3 that all entries of the within-study covariance matrices are regarded as fixed and known. The diagonal entries are obtained in the same way as in the univariate case and are typically given, or can be ascertained, from the published reports of the studies included in the analysis. The within-study correlations are not generally available in this way. It is perhaps partly because of this difficulty that multivariate meta-analysis has primarily found applications in diagnostic testing and IPD meta-analysis so far; in the former the within-study correlations can safely be assumed to be zero, and given IPD one can usually fit the desired model or models and extract the within-study covariance matrix in a routine way. Jackson *et al.* 67 chose to use effects which could be modelled as having zero within-study correlations, and transformed the estimates to provide more interpretable quantities at a later stage, but such an approach is hard to generalize. Riley 13 describes some more widely applicable ways in which this problem can be resolved and we describe these here.

### 6.1. Use an approximate formula

For the special case of mutually exclusive binary outcomes, formulae for the correlations have been derived 68. The existence of such formulae for particular applications is the exception, rather than the rule, however.

### 6.2. Obtain individual patient data

IPD allows us to obtain the entire within-study covariance matrix in each study as noted above, alleviating the reliance on reported information. In more complex modelling situations, bootstrapping methods may be required 10, 22.

### 6.3. Narrow the range of possible values

IPD may be available for some studies. In this situation one solution is to use the within-study correlations derived from IPD studies to inform the likely value of the within-study correlation in aggregate data studies. For example, the average available within-study correlation could be imputed, or sensitivity analyses could be performed by imputing over the range of observed values.

Even without IPD studies, it may be possible to narrow the range of possible values for the unknown within-study correlations. For example, Raudenbush *et al.* 69 used external information for this purpose. For the special situation where multiple relative risks are to be synthesized, Berrington and Cox 70 narrowed the range of possible values for the within-study correlation by calculating lower and upper bounds from the 2 × 2 tables that were available from each study.

### 6.4. Perform sensitivity analyses over the entire correlation range

Where little or no information about the within-study correlations exists, a further option is to perform sensitivity analyses by imputing correlations over the entire range of values (i.e. from −1 to 1), to assess whether and how conclusions depend on the correlation that is imputed. In a Bayesian framework, Nam *et al.* 59 took a similar approach by placing a uniform(−1, 1) prior distribution on the within-study correlation and then assessed whether conclusions are robust to changes in the specification of this prior. Sensitivity analysis for unknown within-study correlations becomes problematic in more than two dimensions. In our examples we only considered non-negative within-study correlations, because of their context, but there are still many more possibilities than we examined.

### 6.5. Use an alternative model that does not require the within-study correlations

An alternative multivariate random effects model for meta-analysis has been proposed which does not require the within-study correlations 71. The data required to fit the model are the same as those needed for a separate univariate analysis of each outcome, which makes it widely applicable. Estimation can however become unstable when the estimated correlation is close to the edge of the parameter space, i.e. −1 or 1. In higher dimensions, such as in our second example where the use of this method might be entertained, there are further constraints on the between-study variance structure. Hence, further investigation into the use of this method for high-dimensional meta-analyses is warranted. This method is now implemented in *mvmeta1*.

### 6.6. Use robust variance estimators

Hedges *et al.* 72 have suggested using robust variance estimates for the treatment effect parameters. Here, a weighted average of the estimated effects is calculated as an estimate of the treatment effect, whose variance is obtained using the residuals and established techniques for evaluating robust variances. We feel that this recent innovation, and variations of it, have the potential to ease the problem of unknown within-study correlations.

To summarize, a variety of approaches are available for handling the common situation where the within-study correlations are unknown. The absence of information about the within-study correlation structure does not entirely prohibit a multivariate approach but this does present very real statistical issues and a consensus about the best approach or approaches has yet to be reached.

## 7. Discussion

We hope that this article will serve to summarize the current multivariate meta-analysis climate, in both theoretical and computational terms, and raise awareness of the type of applications it has found. One vision for the future is that every meta-analysis will eventually be multivariate (unless only a single effect is considered). The concern now is whether or not this is sensible, and is this really what we want? We have found that the multivariate methods have the potential to make a real contribution to meta-analysis, but also that they bring additional complications and issues with them. Our examples are representative of our experiences that multivariate meta-analysis can be helpful in some instances, but are not necessarily so every time. Our third example makes a strong case for those contemplating the use of the multivariate techniques to take even greater care when using them, compared with the ‘old and (perhaps) more reliable’ univariate methods.

We have described the advantages of the multivariate methods but some might reasonably argue that the univariate scenario is still not understood well enough to proceed with the multivariate setting. Conventional univariate meta-analysis requires normal approximations, and replaces variance parameters with estimates, and it is not really understood how many and large the studies must be to make these approximations accurate enough to be appropriate. It is unclear whether the multivariate setting will exacerbate these kind of issues, and if so by how much. There is also the argument that statisticians' energy should go into getting good estimates and standard errors, and avoiding publication biases, rather than developing complex models which may be unnecessary for such simple data structures.

Visual plots are important in any statistical analysis and forest and funnel plots have an established place in univariate meta-analysis. The ‘bubbleplot’, as shown for our first example in Figure 1, is useful for displaying bivariate meta-analyses and two dimensions from higher dimensional meta-analyses. How to attempt to display all aspects of high-dimensional meta-analyses, and produce multivariate funnel and forest plots for example, remains an open question.

It should be noted that multivariate meta-regression 73, where the underlying treatment effects depend on covariates, is a straightforward extension of multivariate meta-analysis 52 and analogous procedures to those described in Section 4.1 have now been implemented in *mvmeta1*. The additional problem of having to estimate the effect of covariates in a conventional univariate meta-regression has been found to require larger sample sizes to perform well 74 and this can also be expected to be the case multivariately.

Alternative multivariate methods are also possible. Multivariate generalizations of descriptive statistics as *I*^2^ 75 are also currently at an early stage of development. When analysing our examples, we focussed on the treatment effect parameters but if some agreement of how *I*^2^ and related statistics should be extended to more than a single dimension could be reached, then we would recommend that these also be provided and interpreted when using multivariate methods. Currently the authors simply calculate *I*^2^-type statistics as the ratio of diagonal entries of the estimated between-study covariance matrix and the sum of this and the usual ‘typical’ within-study variance, obtained from the corresponding univariate within-study variances, but it may be that something more sophisticated than this is both possible and desirable.

Now that we have provided our version of events, we await the testimony of our expert witnesses with considerable interest.
